# Circulating tumor cell-derived preclinical models: current status and future perspectives

**DOI:** 10.1038/s41419-023-06059-6

**Published:** 2023-08-17

**Authors:** Zuzana Kahounová, Markéta Pícková, Stanislav Drápela, Jan Bouchal, Eva Szczyrbová, Jiří Navrátil, Karel Souček

**Affiliations:** 1 Department of Cytokinetics, Institute of Biophysics of the Czech Academy of Sciences, 612 00 Brno, Czech Republic; 2 International Clinical Research Center, St. Anne’s University Hospital, 602 00 Brno, Czech Republic; 3 Department of Experimental Biology, Faculty of Science, Masaryk University, 625 00 Brno, Czech Republic; 4 Department of Clinical and Molecular Pathology, Institute of Molecular and Translational Medicine, Faculty of Medicine and Dentistry, Palacký University and University Hospital, 779 00 Olomouc, Czech Republic; 5 Department of Comprehensive Cancer Care, Masaryk Memorial Cancer Institute, 656 53 Brno, Czech Republic; 6 Present Address: Department of Molecular Oncology, H. Lee Moffitt Cancer Center & Research Institute, Tampa, FL USA

**Keywords:** Cancer models, Metastasis

## Abstract

Despite the advancements made in the diagnosis and treatment of cancer, the stages associated with metastasis remain largely incurable and represent the primary cause of cancer-related deaths. The dissemination of cancer is facilitated by circulating tumor cells (CTCs), which originate from the primary tumor or metastatic sites and enter the bloodstream, subsequently spreading to distant parts of the body. CTCs have garnered significant attention in research due to their accessibility in peripheral blood, despite their low abundance. They are being extensively studied to gain a deeper understanding of the mechanisms underlying cancer dissemination and to identify effective therapeutic strategies for advanced stages of the disease. Therefore, substantial efforts have been directed towards establishing and characterizing relevant experimental models derived from CTCs, aiming to provide relevant tools for research. In this review, we provide an overview of recent progress in the establishment of preclinical CTC-derived models, such as CTC-derived xenografts (CDX) and cell cultures, which show promise for the study of CTCs. We discuss the advantages and limitations of these models and conclude by summarizing the potential future use of CTCs and CTC-derived models in cancer treatment decisions and their utility as precision medicine tools.

## Facts

- Circulating tumor cells play a crucial role in cancer dissemination.
- Circulating tumor cells are rare in the bloodstream.
- In vivo/in vitro models are needed to expand circulating tumor cells for research purposes.

## Open questions

- How accurately do in vitro/in vivo circulating tumor cell-derived models reflect the biology of the original tumor?
- What are the prerequisites and conditions for the successful implementation of these models?
- How can these preclinical models be effectively utilized in the management of patient treatment?

## Introduction

**Fig. 1:**
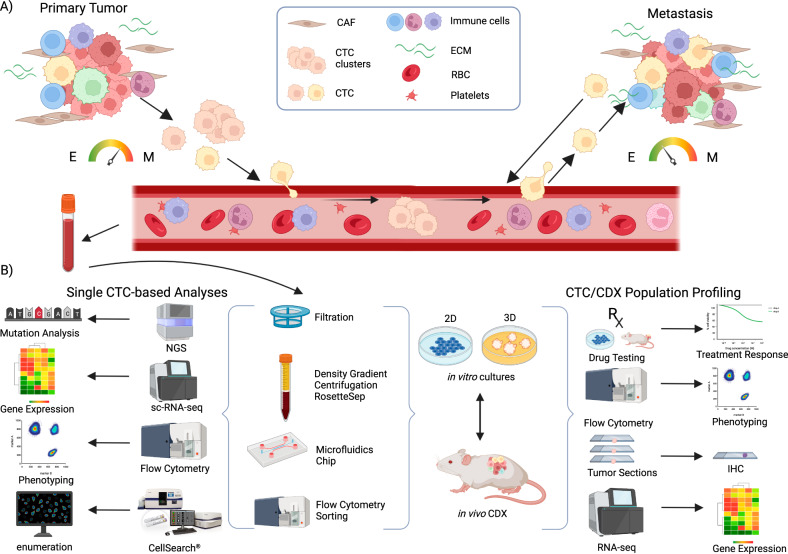
Circulating tumor cells (CTCs) leave the primary tumor as single cells or in clusters, intravasate into the bloodstream and travel through the circulation to the distant site of the body to establish metastasis. **A** At the primary site, epithelial-to-mesenchymal transition (EMT) leading to more mesenchymal (M) phenotype is characteristic for CTCs, whereas in metastatic site, more epithelial (E) phenotype and mesenchymal-to-epithelial transition occurs. Moreover, cancer cells can leave the metastatic site and colonize back its primary tumor site (tumor self-seeding). **B** For establishment of in vivo*/*in vitro preclinical models, viable CTCs are isolated/enriched from peripheral whole blood by several methods (filtration, density gradient centrifugation coupled with negative depletion, microfluidic devices, or flow cytometry). Obtained CTCs can be characterized using various single cell-based technologies or used for establishment of in vitro culture (suspension/adherent/tumoroids) and in vivo CTC-derived xenografts (CDX). In vitro cultures may be used for establishment of CDX and vice versa, developed CDX may serve as a source of material for establishment of in vitro cultures. Both in vivo/in vitro models serve as a source of valuable material for subsequent analyses of CTCs at the single cell level (mutation analyses, sc-RNA-Seq, mass cytometry, microscopy), as well as analysis of bulk population (drug screening and therapy decision making, phenotype analysis using flow cytometry). CAF cancer-associated fibroblasts, RBC red blood cells, ECM extracellular matrix. Created with BioRender.com.

Cancer dissemination is a dynamic process where circulating tumor cells (CTCs) are released from the primary tumors or metastatic lesions into the bloodstream, allowing the cancer to spread. They can also support their tumors of origin, contributing to tumor growth, angiogenesis, and stromal recruitment through a process known as “tumor self-seeding” [1]. While numerous CTCs and CTC clusters are shed as the primary tumor grows, the dissemination process remains highly inefficient, with only a small fraction (approximately 0.02%) of these rare cells successfully completing the metastatic cascade and forming secondary tumors [1–5]. This fact is undoubtedly related to the necessity of multiple changes at many levels, leading to the capacity for intravasation, survival in the systemic circulation, extravasation in target organs, adaptation to the new microenvironment at secondary sites, and finally the emergence of a metastatic lesion (Fig. 1A) [6]. Initially, dissemination was thought to occur predominantly in advanced stages of cancer [7]. However, clinical and experimental observations challenged this notion, revealing that metastases can develop after primary tumor surgery, suggesting that CTCs may be released early in tumor progression and disseminate in a dormant state [7–13]. One of the most significant biological processes contributing to metastatic cascade and cancer progression is the epithelial-to-mesenchymal plasticity (EMP; [14]), which allows cancer cells to change their abilities in response to their environment. This leads to the selection of aggressive tumor cell clones that are capable of numerous adaptations necessary for survival throughout the metastatic cascade [15–20]. The EMP allows a reverse transition from epithelium to mesenchyme (EMT) and from mesenchyme to epithelium (MET). Particularly EMT is associated with the acquisition of stem cell-like properties and plays a crucial role in metastasis development, as it is closely related to the invasiveness, stemness, and chemoresistance of cancer cells [21, 22]. EMT and MET were originally described as processes leading to binary cell states, however, recent studies have demonstrated the existence of several transition states with a hybrid phenotype. Numerous studies have shown that CTCs populations in particular are characterized by this hybrid phenotype and associated EMT/MET heterogeneity (summarized in [18, 19, 23]). For example, while CTCs isolated from the blood of breast cancer (BCa) patients showed both epithelial and mesenchymal markers, this cell type was rarely found in primary tumors [24]. Furthermore, dynamic changes in the pool of CTCs with epithelial and mesenchymal phenotypes were described over the course of anticancer therapy [24].

Dynamic changes in CTCs are not only manifested at the level of changes in their phenotypes, but also in their fluctuating numbers. CTCs are typically present in small quantities, in the order of units to tens per 7.5 ml of whole blood, with significant variations depending on disease stage, cancer type [25, 26], treatment [27], and even on the time of the day [28] or site of blood sampling [29–31]. Over the past two decades, various methods have been developed for the detection, enumeration, and capture of CTCs. When applying the method to isolate CTCs, several factors must be considered, including CTCs yield, maximum blood volume processing capacity, isolation rate, purity of the isolated fraction, viability of isolated CTCs, ability to isolate CTC clusters, and the use of positive or negative selection markers. It is crucial to acknowledge that the phenotypic plasticity of CTCs has fundamental consequences for their detection and isolation. This is particularly true with respect to the epithelial cell adhesion molecule (EpCAM), which is the most used marker for positive selection of CTCs. The first Food and Drug Administration (FDA) approved system for CTCs detection and enumeration is CellSearch® (Menarini Silicon Biosystems) [32]. It detects CTCs based on a combination of epithelial markers EpCAM, CK8, CK18, and CK19 for positive selection of CTCs, and CD45 serving for immune cells elimination. It is used for CTCs quantification in metastatic breast cancer (mBCa), metastatic prostate cancer (mPCa) and metastatic colorectal cancer (mCRC) patients, with cut-off values of ≥5 CTCs for mBCa and mPCa, and ≥3 CTCs for mCRC per 7.5 ml of blood [25, 33–39]. However, EpCAM-based isolation methods do not capture CTCs that lose the epithelial marker due to acquisition of a mesenchymal phenotype [40]. The first medical device to receive FDA clearance for CTCs enrichment in mBCa patients is Parsortix® PC1 system (ANGLE plc.). This semiautomated system enables the collection of CTCs from body fluids based on their size and deformability without the use of any markers for their detection [41]. However, there is currently no universally applicable method that maximizes the number of CTCs while minimizing contamination by other cell types. Therefore, it is essential to find a balance between all these factors to meet the specific requirements of different downstream applications. For a detailed description of specific techniques, their advantages, disadvantages and downstream applications for CTCs recovery by different methods, we refer the reader to the respective articles [42–47].

The correlation between the mass of CTCs and disease progression has become evident with advancements in CTCs detection and counting methods. The number of CTCs has been linked to progression-free survival (PFS) and overall survival (OS) in patients with various types of cancer, serving as a prognostic marker for disease progression and as a predictive marker for treatment response (reviewed in [48]). Although the determination of circulating tumor cells (CTCs) counts has proven to be clinically useful, there is an urgent need to develop experimental and preclinical models derived from CTCs. Such models would address the limitations associated with the low abundance of CTCs in patients’ blood, allow the investigation of tumor spread in relevant models and facilitate preclinical studies, including the evaluation of the effects of new drugs. In this comprehensive review, we provide an overview of recent advancements in the development of CTC-derived xenografts and CTC-derived cell cultures (Fig. 1B). We highlight the advantages and limitations of each approach for disease modeling, drug testing, and improving of anticancer therapy.

## Preclinical models of CTCs

### CTC-derived xenografts

Patient-derived xenografts (PDXs), established by engrafting pieces of human tumor tissue into immunocompromised mice, represent a very useful preclinical model for more faithful disease modeling and a step towards precision medicine. Obtaining biopsy material from both the primary tumor and metastases is an invasive and, in some types of tumors, challenging procedure. CTCs, on the contrary, are accessible through less invasive routine blood draw, which also allows and facilitates repeated sampling for easier disease monitoring. CTC-derived xenograft (CDX) models offer a representative molecular snapshot of the disease as they serve as surrogates of otherwise unobtainable metastatic tissues. Due to the significant differences in individual cancer types, the following section is organized according to the types of tumors in which CDX derivation has been described (see Table 1 for details).

**Table 1:** Overview of published studies with CDXs.

Cancer type	Isolation method	Blood volume	Strain	Implantation	Success rate	Latency	References	Notes
Small cell lung cancer	CTC-iChip^neg^ device	15-20 ml	NSG	s.c.	16/42	115 days	[57]	
Small cell lung cancer	RosetteSep™ human circulating epithelial tumor cell cocktail	10 ml	NSG	s.c. in flanks	4/6	4 months (2.4–4.4)	[55]	>400 CTCs per 7.5 ml
Lung cancer	RosetteSep™ CTC enrichment cocktail containing anti-CD36	10 ml	NSG	s.c. in flanks	17%	Up to 1 year	[58]	CTC count (7.5 ml) <50 = 2/146; >50 = 35/71
Non-small cell lung cancer	RosetteSep™ human circulating epithelial tumor cell cocktail	10 ml	NSG	s.c. in flanks	1	95 days	[40]	From post-radiotherapy CTCs
Non-small cell lung cancer	RosetteSep™ cocktail	7.5 ml	NSG	Interscapular fat pad	4/55	100, 200, 116, 100 days	[60]	CTCs injected 3500, 35,330, 1102
Prostate cancer	RosetteSep™ CTC enrichment cocktail containing anti-CD36™	7.5	NSG	Interscapular region	0/15	10 months	[53]	CTC number—median 230, range 0–18,389
Prostate cancer	Diagnostic leukapheresis + RosetteSep™ CTC enrichment cocktail containing anti-CD36	2/3 of DLA product	NSG	Interscapular region	1/7	165 days	[53]	CTC number 0–19,988 (median 698), 10 mg testosterone capsule in mice flanks
Prostate cancer	RosetteSep™ human circulating epithelial tumor cell cocktail	10 ml	NSG	s.c.	1	NA	[109]	100–10,000 CTCs
Triple-negative breast cancer	Density gradient centrifugation protocol (Histopaque) in SepMate™ tubes	7.5	NUDE	s.c.	1	5 months	[50]	CTC count = 969, patient with highest CTC number selected
Triple-negative breast cancer	FACS sorting (CD45^−^/CD34^−^/CD105^−^/CD90^−^/CD73^−^ cells)	8 ml	NSG	Intracardiac	1/ 3		[51]	Liver metastasis
Metastatic breast cancer	Hematopoietic cells depletion	7.5	NSG	Femoral tumor cavity	6/110	6–15 months	[49]	CTC count <1000 = 0, CTC count >1109 = 6
Melanoma	RosetteSep™ depletion cocktail	7.5	NSG	s.c.	6/47	1 month 2.5 months	[61]	

Summary of the main characteristics of studies describing generation of CDXs.

*s.c.* subcutaneous, *NA* not analyzed.

#### Breast cancer

The first CDX model was established and described by Baccelli and colleagues 10 years ago using CTCs from mBCa patients implanted into the bone marrow of mice [49]. Further dissemination of these cells to the liver and lungs confirmed the presence of metastasis-initiating cells among the CTCs. First CDX of triple-negative BCa (TNBC) was generated from patients with CTCs count more than 900 per 7.5 ml of blood, as estimated by EpCAM-based CTC counting using CellSearch® system analysis in a parallel blood sample [50]. The developed CDX displayed common molecular and immunohistological features with the primary tumor and highlighted the importance of Wnt signaling pathway for tumor progression. Another study developed and described a CDX model of liver metastasis of TNBC, which was used for identification of CTC-associated liver metastasis signature [51]. Intracardiac injection of isolated CTCs from two patients without liver metastases led to the development of liver metastases in mice. However, when CTCs were obtained from TNBC patients with liver metastases, no liver metastases developed in the CDX model, demonstrating the existing limitations of this approach. Klotz and colleagues established CDX for the study of brain metastatic activity of luminal BCa. They used four GFP-Luc-labeled CTCs lines previously derived from patients with luminal BCa, for monitoring the development of metastases in mice for at least 5 months and revealed driver genes for brain metastases [52].

#### Prostate cancer

Faugeroux and colleagues were the first who developed a prostate cancer (PCa) CDX model [53]. For successful generation of the CDX, they used leukapheresis followed by hematopoietic cells depletion and increased the yield of CTCs to approximately 20,000. The time to xenograft growth was about twice as short than in other studies, which increases the chances for the translation of the findings to the patient. Importantly, a few foci of neuroendocrine (NE) cell markers were detected in the primary tumor. These NE markers were highly expressed in CDX, indicative of the emergence of AR-null, neuroendocrine-positive phenotype of CDX which developed in mice. Moreover, CDX precisely recapitulated the patients’ response to the docetaxel and enzalutamide therapy in vivo. Finally, they established CDX-derived cell line, which had phenotypic, functional, and genetic characteristics comparable to original CDX and may serve as a valuable model for testing of new treatments.

#### Lung cancer

Several studies describe CDX models of small cell lung carcinoma (SCLC), as this type of primary tumor is known to shed high numbers of CTCs [54]. Hodgkinson and colleagues established CDX from CTCs from SCLC patient; CDX had genomic characteristics comparable to isolated CTCs [55]. Established CDXs recapitulated responses of donor patients to cisplatin or etoposide, which are standardly used for the treatment of SCLC. Comparison of SCLC CDX models from chemotherapy-naive and chemotherapy-resistant patients suggested existence of a predictive genomic signature for inherent resistance to the conventional chemotherapy. Moreover, the response of CDXs to therapy closely mirrored the overall survival of the patients. Patient’s single CTCs, pool of ten CTCs, and pool of white blood cells were isolated from patients with SCLC, subjected to WGA and CNA and compared to CDXs to inspect potential phenotypic changes [55]. Results of analysis of single CTCs strongly correlated with the corresponding CDX, although some heterogeneity in original CTCs was also detected. In the follow-up work, ex vivo cultures from several CDXs were established [56]. These CDX-derived cells grew as floating clusters in non-adherent conditions ex vivo, and in a short term (4–5 weeks), the cultivation did not lead to substantial clonal selection. Since the establishment of this ex vivo culture was quite efficient (16 out of 20), it provides a useful platform for drug screening. Moreover, genetic manipulation using lentiviral transduction was successful in this study, opening another window for functional tests of druggable targets [56].

Human SCLC xenograft models derived from primary tumor biopsy (PDXs) and CTCs (CDXs) were compared [57]. Whole-exome sequencing confirmed that the paired CTCs collected at the time of the biopsy share the same genomic features as the sampled solid tumor. Moreover, SCLC PDX models retained a stable genome and maintained their somatic alterations in serial passages, faithfully recapitulating SCLC patient tumors at the time of model generation. Both models harbored inactivating alterations of *TP53* and *RB1*, well-known mutations for SCLC. Lastly, the CDX models derived from serial sampling of a patient in the progressive phase, treated with several drugs prior to CDX generation and not responding to the therapy anymore, also reflected the development of resistance to the treatment in vivo.

In another study of SCLC, CDXs from various stages of the disease and therapy phases were generated with a success rate of about 17% after subcutaneous injection [58]. Multiple types of analyses deeply characterized the intra- and inter-tumor heterogeneity of CDXs, classified cancer subtypes, and even identified new subtypes of the SCLC based on molecular markers (ASCL1, NEUROD1, POU2F3, and ATOH1). Lastly, intratumoral heterogeneity was evident at the level of neuroendocrine markers ASCL1/NEUROD1 and three groups were classified in multiple CDXs (ASCL1^+^/NEUROD1^−^, ASCL^+^/NEUROD1^+^, ASCL1^−^/NEUROD1^+^).

Another CDXs from CTCs from SCLC were derived by Stewart and colleagues [59]. Comparison of platinum-sensitive and platinum-resistant SCLC CDXs using single-cell RNA-seq revealed increased intratumoral heterogeneity, with the onset of chemoresistance and appearance of resistance-associated populations of cells defined by established drug resistance gene signatures. When platinum sensitive CDXs were treated with cisplatin until relapse, increased intratumoral heterogeneity and variations in expression of the therapeutic targets within the same patient were detected. Increased intratumoral heterogeneity was confirmed when CTCs from patients before, during, and after platinum relapse were analyzed.

Together five CDXs were so far established from non-small cell lung cancer (NSCLC) models. Morrow and colleagues established CDX from CTCs obtained from blood of patient with metastatic NSCLC after brain radiotherapy, but not from CTCs obtained from the same patient before therapy [40]. Interestingly, parallel analysis of CTCs with CellSearch® detected 4 EpCAM^+^/CK^+^ CTCs in patient blood before therapy, but no EpCAM^+^/CK^+^ CTCs post-therapy. There were more than 150 CTCs in post-therapy blood sample with epithelial CK^+^/vimentin^–^ (23%), mesenchymal CK^–^/vimentin^+^ (30%), and mixed phenotype CK^+^/vimentin^+^ (47%) CTCs. In vivo propagated CDX was resistant to treatment with cisplatin and pemetrexed, the similar combination of therapeutics used in patient. Next, Tayoun and colleagues established 4 CDXs from patients with NSCLC after implantation of CTC-enriched fraction in interscapular fat pad [60]. Interestingly, the success of CDX establishment seemed to be independent of number of CTCs implanted, since they established CDX from 3500, 330, 1102, but also only 35 CTCs.

#### Melanoma

Only a few studies have demonstrated the generation and utilization of CDX from melanoma CTCs. A CDX developed from a patient with highly aggressive BRAF-V600E melanoma unresponsive to targeted therapy resembled the patient’s histological features [61]. In mice bearing this CDX, lung micrometastases were detected, and after primary tumor excision, liver macrometastases appeared, showing that the metastatic tropism of the CDX was the same as in the patient. Finally, the CDX failed to respond to the same drug treatment as did the patient. Vishnoi and colleagues isolated CTCs from melanoma patients and developed CDXs after intracardial injection of CTCs [62]. They collected long bones and organs usually affected by metastatic melanoma, and although no macrometastases were detected after 6 months using standard IHC, staining for specific markers showed presence of human melanoma cells in multiple murine organs. Comparison of bone marrow-resident tumor cells versus CTCs from this model identified protein ubiquitination as an important regulatory pathway in tumor cells, as inhibition of a specific deubiquitinating enzyme USP7 decreased systemic micrometastases.

#### Prerequisites and limitations of CDX models

One significant advantage of CDX models is their ability to propagate CTCs and generate a significantly higher number of cells compared to the original CTCs obtained from a patient’s blood. This expansion can be performed repeatedly as the disease progresses in a single patient. Recently, the use of CDX models has been described for the identification of genes associated with various steps in the metastatic cascade [63]. In one study, CTCs isolated from a breast cancer (BCa) patient were expanded in vitro and injected into a mammary fat pad to establish a CDX model and by employing a genome-wide loss of function CRISPR screen, several genes associated with different stages of metastasis were identified. These included genes associated with the formation of CTC clusters (IL18R1, ITGA2, CSNK1A1L, and CSNK2A2), genes associated with intravasation (PLK1), and genes implicated in brain-specific metastasis (HDAC and Rho GTPases).

On the other hand, there are several limitations to keep in mind when working with CDX models. The latency can vary from several months to almost one year (Table 1). This time requirement negatively affects the use of CDX models in drug testing in precision medicine and particular patient treatment selection/modification, as progressing disease does not allow waiting months for CDX development. Moreover, housing of immunodeficient animals for many months brings additional considerable costs to these models. The efficiency of successful CDX establishment differs in various cancer types, and even within one cancer type in various studies. The crucial factor for CDX establishment seems to be the number of CTCs isolated from the patient. Implanting less than 1000 mBCa CTCs resulted in no xenograft generation in 15 months, whereas implanting at least 1109 CTCs resulted in a successful xenograft generation [49]. In PCa, CDX was successfully established only when 19 988 CTCs (obtained by leukapheresis) were implanted in a mouse [53]. This confirmed a previous study showing that apheresis leads to higher CTCs yields in PCa patients—the average CTC yield from 7.5 ml of peripheral blood assessed by CellSearch® was 167 CTCs, whereas the apheresis processed 59.5 ml of blood with an average yield of 12 546 CTCs [64]. Similarly, the use of diagnostic leukapheresis (DLA) for CTC isolation in mBCa and mPCa patients significantly increased the yield of CTCs compared to CellSearch® [65].

Based on the information provided, it becomes evident that determining the number of CTCs in each sample prior to the generation of CDX models is crucial. It is important to note that the quantification of CTCs relies heavily on the methodology employed. The CellSearch® system, for instance, detects CTCs by targeting the epithelial marker EpCAM, thereby excluding EpCAM-negative CTCs from the analysis. Remarkably, a CDX model was successfully established from a patient whose CTCs were undetectable using the CellSearch® method [40]. The resulting CDX exhibited positive expression of mesenchymal markers, aligning with previous findings suggesting that CTCs can evade EpCAM-based detection methods due to undergoing epithelial-mesenchymal transition (EMT) [66]. Consequently, it is advantageous to employ CTC isolation and enrichment techniques that are not reliant on selective epithelial or mesenchymal markers.

Another factor influencing the development of CDX is the site of injection/implantation of isolated CTCs. Subcutaneous implantation is a widely used method (Table 1). The importance of implantation site was discussed by Dasgupta and colleagues—implantation into a solid tissue (fat pad, flanks) facilitates establishment of a solid tumor without the need to survive in circulation, extravasate, and establish the tumor from a single cell without support from other tumor cells [67]. On the other hand, the injection of CTCs directly into the bloodstream implies the possibility of finding a microenvironment that is comparable to the microenvironment from which they originate or to which they can adapt. For example, an intracardiac injection was used for the generation of CDX liver metastasis model [51]. Since CTCs are responsible for metastases development, a recently introduced murine model of bone metastasis initiated by injection of cancer cells via caudal arteries may also be applicable for CDX generation, although the monitoring and excision of a developed tumor might be more difficult compared to a subcutaneous model [68].

In summary, CTC-derived xenografts are a valuable model for disease monitoring, searching for candidate genes and/or mutations associated with disease progression or response to treatment, and studying intratumoral heterogeneity. However, the low efficiency and long latency of tumor development in mice currently disqualifies this method for precision medicine, which requires flexible management of each patient’s treatment strategy. This would require greater robustness, higher CDX establishment efficiency, and shorter time to xenograft generation.

### In vitro CTC-derived cultures

The CDX generation shows several limitations, as described in the previous chapter. Therefore, the research is focusing also on the seemingly less challenging derivation of in vitro cultures from CTCs. In this chapter, we discuss various aspects important for CTCs cultivation, e.g., short- vs. long-term in vitro expansion, normoxic vs. hypoxic conditions, 2D vs. 3D conditions, or adherent vs. non-adherent cultures. Due to specific conditions in individual cancer types, the following section is organized according to the types of tumors from which CTCs cultivation has been described (see Table 2 for an overview).

**Table 2:** Overview of published studies with in vitro CTC cultivation.

Cancer type	Isolation method	Efficiency	Cultivation conditions			Tumorigenicity	References	Notes
			Hypoxia	Non-adherent/adherent	2D/3D			
Breast cancer	CTC-iChip	6/36	Hypoxia	Non-adherent	Tumoroids	3/6 tumorigenic	[70]	
Breast cancer	RosetteSep™	1/50	Standard	Mixed	2D	Tumorigenic	[71]	
Breast cancer	Ficol-Paque density gradient	12/12	NS	Attached to leukocyte layer	–	NA	[72]	CD45^+^ leukocytes included in culture
Breast cancer	RosetteSep™ CTC enrichment cocktail containing anti-CD56	36/48	Hypoxia for 1 week, then standard	Non-adherent	–	NA	[97]	Nanoemulsions in culture media
Breast cancer	Ficoll-Hypaque gradient centrifugation	1/16	–	Adherent	2D	Tumorigenic	[74]	0/16 bowel cancer, 0/18 stomach cancer
Non-small cell lung cancer	Microfluidic Herringbone-Chip and EpCAM-coated and EGFR-coated immunomagnetic microbeads	2/89	Hypoxia followed by standard	Non-adherent followed by adherent	2D	Tumorigenic	[82]	Only 1 maintained more than 6 months in culture
Non-small cell lung cancer	RosetteSep™ Human CD45 depletion cocktail	9/70	Hypoxia	Adherent	2D	NA	[98]	Short-term culture (40 days)
Small cell lung cancer	Density gradient centrifugation	3/30	Standard	Adherent	2D	NA	[79]	
Small cell lung cancer	RosetteSep™ CTC enrichment cocktail	18/22	Standard	Adherent	Spheroids	NA	[81]	Cultivation on binary colloidal crystals
Early-stage lung cancer	CTC-capture microchip with EpCAM-based detection	14/19	Standard	–	3D	NA	[84]	Coculture with CAF on collagen/matrigel
Colorectal cancer	Density centrifugation with negative selection form CD45	1/4	Standard	–	Tumoroids	NA	[110]	CTC isolated from PDX mouse model
Colon cancer	RosetteSep™ human circulating epithelial tumor cell enrichment cocktail	2/71	Hypoxia followed by standard	Non-adherent	Spheroids	Tumorigenic	[86]	CTC count of ≥300.
Metastatic colorectal cancer	RosetteSep™ human circulating epithelial tumor cell enrichment cocktail	3/35	Standard	Suspension	Spheroids	Tumorigenic	[88]	Successful establishment only from stage IV patients without neo-adjuvant therapy
Pancreatic cancer	Size-based microfluidic CTC isolation with labyrinth	3/10	Standard	Adherent	Spheroids	Tumorigenic	[90]	Originally adherent cultures on fibronectin-coated surface, followed by cultivation as non-adherent spheroids
Pancreatic cancer	RosetteSep™ CTC enrichment cocktail kit	36/41	–	–	Tumoroids	NA	[91]	Cells seeded onto a binary colloidal crystal substrate containing silica and polymethyl methacrylate
Prostate cancer	RosetteSep™ human CD45 depletion cocktail	1/17	Standard	–	Organoids		[77]	CTC count more than 100/8 ml blood
Prostate cancer	Diagnostic leukapheresis + RosetteSep™ Human CD45 depletion cocktail	14/40	Standard	–	Tumoroids	NA	[78]	6–8 weeks is culture until proliferation stalled, 2 cultures more than 6 months, 1 culture gave rise to stable cell line
Endometrial, prostate, ovarian, gastric cancer	MetaCell® filtration	Various	Standard	Adherent on filter	–	NA	[111–114]	Short-term (2 weekS) and long-term (6 months) efficiency of CTC capture 52% PCa, 75% endometrial, 59% gastric, 65% ovarian
Gastroesophageal cancer	RosetteSep™ CTC enrichment cocktail with anti-CD36	2/23	Hypoxia	Adherent/non-attached	–	Tumorigenic	[115]	Long-term culture over 1 year
Lung, colon, pancreatic cancer	Ficol-Paque density gradient	5/5 lung 5/7 colon 13/13 pancreas		Adherent		Tumorigenic	[73]	Short-term culture, CTCs attached to CD45^+^ cells

Summary of the main characteristics of studies describing short- or long-term in vitro culture of CTCs.

*CAF* cancer-associated fibroblasts, *NA* not analyzed, *NS* not specified.

#### Breast cancer

The first short-term in vitro CTC-derived cultures were established in 2013 by Zhang and colleagues from BCa patients with metastatic disease [69]. Using in vivo models, a subpopulation of CTCs responsible for brain metastasis was identified. Yu et al. established CTC-derived cell lines from six patients with estrogen receptor-positive mBCa, which were able to proliferate for more than 6 months in vitro [70]. Non-adherent conditions were critical for the establishment of in vitro cultures because adherent cells became senescent after several divisions. These cultures were further successfully used for in vitro drug screening. Another cell line established from a metastatic ER^+^ BCa patient grew under standard cultivation conditions in both adherent and non-adherent fractions [71]. The cells displayed a mixed epithelial–mesenchymal morphology but expressed predominantly markers associated with epithelial phenotype. Analysis of copy number alterations showed that the established cell line largely resembled originally isolated CTCs. In vitro testing showed sensitivity of this cell line to a CDK 4/6 inhibitor, which suggests that the patient could benefit from such treatment.

Co-cultivation of CTCs with other cell types led to successful cultivation of several CTC-derived cell cultures. A 100% success rate in short-term cultures from metastatic BCa cells was associated with initial seeding of CTCs together with CD45^+^ leukocytes, which were subsequently eliminated from the culture [72]. Only CTCs associated with CD45^+^ leukocytes were able to propagate for more than 30 days in vitro. This way of cultivation was later successful also for lung, colon, and pancreatic cancer [73]. Similarly, another BCa CTC-derived cell line was successfully established when the whole PBMC fraction after red blood cells depletion was seeded and propagated in vitro [74]. Additionally, Khoo and colleagues used co-culture of CTCs with white blood cells on a special chip in hypoxic conditions [75]. Recently, in vitro culture of CTCs with the addition of nano emulsions containing fatty acids and lipids, which increased the proliferation of CTCs, was described [76]. Interestingly, patients, whose CTCs were able to propagate in vitro for more than 23 days (cut-off value for successful CTC culture), had a shorter time to disease progression.

#### Prostate cancer

Nowadays, only a few studies describe successful establishment of CTC-derived in vitro cultures from PCa patients. Gao and colleagues introduced a protocol for organoid generation from metastatic PCa patients—they established six organoid cell lines from biopsies and one cell line from isolated CTCs [77]. When these cell lines were transplanted into immunocompromised mice, the resulting tumors recapitulated the histology of the patient’s original tumor. To increase the yield of CTCs, and concomitantly to increase the success rate of establishment of ex vivo culture, Mout and colleagues employed diagnostic leukapheresis (DLA) for CTC enrichment [78]. DLA from 5 liters of blood led to the median yield of 64 CTCs/ml compared to 2.5 CTCs/ml in peripheral blood samples pre-DLA. Whole genome sequencing (WGS) was performed on multiple single cells obtained from in vitro cultivated PCa CTC-derived tumoroids to uncover the heterogeneity of CTCs and tumoroids [78]. The established organoid cell line was treated with enzalutamide and taxane and showed partial resistance to enzalutamide, like the donor patient.

#### Lung cancer

The first CTC-derived cell lines from SCLC patients were established by Hamilton and colleagues who maintained them in serum-free medium under standard cultivation conditions [79]. Later, another two cell lines were established and used for testing of chemotherapeutic drugs [80]. In 2020, Lee and colleagues described an efficient method for CTCs ex vivo expansion using cultivation on specific biomimetic material called binary colloidal crystals [81]. Cultivation on this substrate led to the formation of spheroids within 14 days. These spheroids were used for in vitro drug screening with cisplatin and etoposide and the results showed that the sensitivity of the established cell lines to these drugs was consistent with the clinical response of the donor to cisplatin/etoposide treatment.

CTC-derived cell lines from NSCLC were established after microfluidic-based immunomagnetic isolation [82, 83]. Initially, hypoxic conditions were used, and once proliferating, the CTC-derived cell line was maintained under standard conditions. Zhang and colleagues used a microfluidic CTC chip for both capture and expansion of CTCs from early-stage lung cancer patients [84]. The most suitable condition for expansion was 3D growth of CTCs combined with cancer-associated fibroblasts (CAFs) co-culture. Expanded cells were successfully used for immunofluorescence and sequencing analyses.

#### Colon cancer

Establishing in vitro CTC-derived cultures from colorectal cancer patients is even less successful than from BCa or PCa. Possible explanations for this could be the fact that there is a much lower number of CTCs in the blood of colon cancer patients, or the low volume of blood tested [85]. The first colon cancer CTC-derived cell line was described by Cayrefourcq and colleagues [86]. Non-adherent CTCs ex vivo culture was established from a metastatic patient with 302 CTCs per 7.5 ml of blood, while attempts from other patients with lower CTCs numbers were unsuccessful. Single-cell-based transcriptome analysis of stable colon cancer CTC-derived cell line demonstrated phenotypic stability over 13 months of in vitro cultivation [86]. In the follow-up, another 8 cell lines from the same patient were established during disease progression and drug treatment and were compared to the cell line obtained at diagnosis [87]. Genotypic analysis showed that the cell lines were of polyclonal origin. Furthermore, all cell lines displayed a partial EMT phenotype and stemness features, which are phenotypes favoring the establishment of distant metastases.

Another three cell lines were established from chemotherapy naïve patients with metastatic colorectal cancer. These cell lines displayed properties of cancer stem cells and contained multipotent cells able to differentiate into all three main intestinal lineages. No cell lines could be established from patients with lower disease stages or treated with chemotherapy [88].

#### Pancreatic cancer

Short-term cultures of CTCs from PDAC were established by Arnoletti and colleagues [89]. Ex vivo culture of CTCs led to formation of spheroids within 7 days, in which CTCs recruited several immune cell types including myeloid fibroblasts. Formation of these heteroclusters in patients may be associated with immune resistance and metastatic progression. Another pancreatic CTC-derived cell lines were established from 3 out of 10 non-treated patients with locally advanced pancreatic cancer [90]. All three cell cultures displayed a mixed epithelial–mesenchymal phenotype and were tumorigenic when injected into flanks of NOD-SCID mice. Recently, tumoroids derived from CTCs from PDAC were established using eSelect biomimetic cell culture system [91]. Drug sensitivity of these ex vivo cultivated CTCs correlated with clinical outcomes of donor patients.

#### Prerequisites and limitations of in vitro CTCs cultures

Since the number of CTCs obtained from patients’ blood is very limited, in vitro expansion of these cells may help to increase the amount of material for subsequent biochemical, molecular, or drug treatment analyses. Furthermore, CTCs expanded in vitro may also be used for CDX generation, where implantation of higher cell numbers may increase the success rate of CDX establishment. Therefore, it is crucial to work on improvement of in vitro cultivation conditions.

Of note, the efficiency of establishment of CTC-derived in vitro cultures varies widely between cancer types and laboratories (Table 2). On the other hand, compared to CDX models, which take months to establish, CTCs cultures can be introduced relatively faster and even short-term cultures (within a few weeks) can be successfully used for drug testing. As obvious from published studies (summarized in Table 2 and discussed below), there are many variable factors in establishing and/or maintaining CTC-derived in vitro cultures, which affect the global characteristics of each new cell line. There is also a great variability in the composition of cultivation media, ranging from very basic to very rich in various growth factors, as summarized elsewhere [92].

Hypoxia and HIF signaling are important factors affecting many steps in the metastatic cascade—immune evasion, migration and invasion, establishment of premetastatic niche, or survival of cancer cells in distant sites (summarized in [93]). CTCs showed a distinct response to hypoxia in vitro, and increased aggressiveness in xenograft models [94]. Intratumoral hypoxia was shown to support intravasation of CTC clusters [95]. Moreover, HIF signaling activated in response to hypoxia promotes expression of stemness-related genes (Oct4, Sox2, Nanog), leading to the support of adult and embryonic stem cells, induced pluripotent stem cells, and cancer stem cells (reviewed in [96]). Therefore, it is reasonable to consider hypoxic conditions for the establishment of CTC-derived in vitro cultures, at least for a short period of time, to initiate proliferation [70, 86, 97, 98].

Another variable factor in establishing CTC-derived in vitro cultures is the choice of 2D or 3D conditions. Setting up of adherent 2D conditions is without debate technically easier. However, CTCs under 2D conditions may undergo senescence after several divisions [70]. Still, several CTC-derived cell lines were successfully established as adherent [79, 81, 90]. Recognizing the limitations of standard adherent cultures in vitro, there are efforts to create 3D cultures from CTCs because they better reflect the original tumor condition. Successfully established 3D tumor spheroids (tumoroids) derived directly from CTCs or CDXs could be used for drug screening, similar to the recently described PDX-derived tumoroid platform for BCa [99]. Drug screening with tumoroids derived from CTCs of a patient with PDAC showed resistance to 5-fluorouracil, oxaliplatin, paclitaxel, and irinotecan, which was consistent with clinical situation in the patient, whose disease progressed despite treatment with FOLFIRI (folinic acid + 5-fluorouracil + irinotecan) [91]. On the other hand, tumoroids were sensitive to erlotinib, and combination of erlotinib with gemcitabine led to therapeutic response. This illustrates the promising use of CTC-derived tumoroids in therapy decision and personalized medicine. However, both 2D and 3D culture conditions lack interaction with other components of the tumor microenvironment. Several co-cultures with various cell types (tumor-associated white blood cells [75], CD45^+^ cells [72, 73], cancer-associated fibroblasts and extracellular matrix (ECM) [84]) were already described to support successful establishment of CTC-derived cultures.

In summary, CTC-derived cultures are another valuable source of material for the study of primary tumors and metastases. Comparison of BCa patient-derived CTCs cultivated in vitro, and matched primary tumors revealed that a large proportion of mutations identified in cultured CTCs were detected also in matched primary tumors [100]. Moreover, CTCs are shed from the whole tumor and therefore reflect its original heterogeneity. In advanced stages of the disease, CTCs originate also from metastases from different sites, reflecting the biology of metastatic disease.

### In ovo CTC-derived models

As discussed in previous chapters, introduction of both in vivo and in vitro models of CTCs is still challenging, requires large time and financial costs, and has relatively low efficiency. Therefore, alternative models to those mentioned above are being developed.

One alternative model is the in ovo assay using chorioallantoic membrane (CAM). The CAM assay is already established in cancer research and is used to assess many aspects of cancer, such as tumor growth, vasculature, invasion, metastatic potential, genomic instability, mutations, or epigenetic reprogramming (summarized in [101]). In 2022, Pizon and colleagues published for the first time the inoculation of cultured tumorspheres from isolated circulating cancer stem cells onto CAM to generate CDX [102]. Tumors on CAM were successfully established from five out of ten BCa patients. Histologically, CAM-induced tumors were confirmed to be comparable to the patients’ original tumors. Importantly, it took only 8 days from inoculation to tumor formation, which is much faster than xenografts formation in immunocompromised mice. Rousset and colleagues generated CAM tumor xenografts from CTCs of BCa, PCa and lung cancer patients with engraftment efficiency of 50% in BCa, 67% in PCa, and 62% in lung cancer cohort [103]. Using next generation sequencing for one selected patient, similar constitutional genomic homozygous polymorphism in *TP53* was found in the original patient’s tumor, in isolated cell-free DNA, fresh CTCs, and in two in ovo tumors generated by CTCs engraftment in CAM.

The CAM assay is a rapid model suitable for studying metastatic cascade and drug treatments [104]. Moreover, it provides an alternative model to in vivo studies, reflecting the 3Rs guidelines. Therefore, these pioneering studies with CTCs need to be further developed.

## Utilization of preclinical models derived from CTCs

Numerous studies described clinical validity of CTCs counts in cancer screening, as a predictor in localized tumors and independent prognostic factor in metastatic cancers (summarized in [105]). Molecular characterization of these models informs us about the mutation status or de novo mutations in CTCs, which gives the opportunity to target these mutations in order to affect CTCs population. How can results obtained with preclinical in vitro and in vivo models be used in clinical practice? One of the goals is to use these models in personalized medicine to uncover druggable mutations or drug resistance mechanisms resulting in tailoring patient’s specific treatment. Yu and colleagues established several CTC-derived in vitro cultures from mBCa patients [70]. Mutation screening uncovered de novo mutation in CTCs acquired during the therapy, and drugs targeting these mutations were then successfully tested on in vitro cell lines. Franken and colleagues combined whole-exome sequencing and RNA profiling of CTCs and identified druggable mutations in a patient with progressing BCa [106]. Then, short-term CTC-derived in vitro cultures were treated with selected drugs and based on these results, therapy was changed in the patient, from whom CTCs were isolated. This led to dramatic drop in CTCs counts in the patient and stabilization of the metastatic disease. This nicely illustrates how CTC-based analyses may be used in precision medicine and in making patient-tailored treatment decisions.

Similarly, CTC-derived xenografts (CDXs) may serve as a robust model for drug screening and molecular characterization. Several studies described similarity between CDX and patient’s original tumor—grown CDXs have usually comparable histological characteristics and key genetic characteristics and importantly, display comparable response to drugs as patient’s original tumor (e.g. [40, 55, 61]. For SCLC, several CDXs were established from CTCs of one patient harvested in various time points during disease progression [57]. These CDXs recapitulated the evolution of drug sensitivity of the patient’s original tumor and are therefore a suitable model to study the acquisition of resistance. Derivation of biobank of CDXs from patients in various stages of disease and even more from the same patient before and during therapy was described for SCLC [58]. This suggests the direction for the use of CDXs in personalized medicine. However, CDXs derived from patients with non-metastatic disease are currently lacking, as the long latency of xenograft development and non-standardized protocols for xenografts establishment are still hurdles to overcome.

## Conclusions and future perspective

CTCs play a significant role in the process of cancer dissemination and their analysis provides valuable insights into the metastatic cascade. Numerous efforts have been made to enhance the detection and capture of CTCs and to improve the yield of isolated CTCs. For instance, the CellCollector®, a sterile steel wire coated with EpCAM antibody, has been introduced to collect CTCs in vivo from the patients’ cubital vein, overcoming the limitations associated with the small blood volumes typically used for CTCs isolation and resulting in increased CTCs yield [107]. Furthermore, a recently described novel whole blood purifier, inspired by hemodialysis, utilizes a specially modified spiral-like glass tube coated with anti-EpCAM to capture both individual CTCs and CTC clusters [108].

As detection and isolation techniques advance, along with the establishment of preclinical in vivo and in vitro models derived from CTCs, there is growing interest in utilizing CTCs as a source of information for disease status, therapeutic response, and treatment decision-making. However, important considerations need to be carefully addressed for both CDXs and CTC-derived cell cultures. These include understanding how well they represent the patient’s original tumor, their robustness and expandability, and the speed at which these models can be generated to bring the benefit for patients. These fundamental questions require further exploration and answers before these preclinical models can be effectively utilized to predict treatment responses and guide individualized treatment decisions in clinical practice.
