# Correction: Circulating tumor cell-derived preclinical models: current status and future perspectives

**DOI:** 10.1038/s41419-024-06825-0

**Published:** 2024-07-02

**Authors:** Zuzana Kahounová, Markéta Pícková, Stanislav Drápela, Jan Bouchal, Eva Szczyrbová, Jiří Navrátil, Karel Souček

**Affiliations:** 1 Department of Cytokinetics, Institute of Biophysics of the Czech Academy of Sciences, 612 00 Brno, Czech Republic; 2 International Clinical Research Center, St. Anne’s University Hospital, 602 00 Brno, Czech Republic; 3 Department of Experimental Biology, Faculty of Science, Masaryk University, 625 00 Brno, Czech Republic; 4 Department of Clinical and Molecular Pathology, Institute of Molecular and Translational Medicine, Faculty of Medicine and Dentistry, Palacký University and University Hospital, 779 00 Olomouc, Czech Republic; 5 Department of Comprehensive Cancer Care, Masaryk Memorial Cancer Institute, 656 53 Brno, Czech Republic; 6 Present Address: Department of Molecular Oncology, H. Lee Moffitt Cancer Center & Research Institute, Tampa, FL USA

**Keywords:** Cancer models, Metastasis

Correction to: *Cell Death and Disease* 10.1038/s41419-023-06059-6, published online 17 August 2023

In this article a grant project number has been given erroneously.

This work was supported by funds from the Czech Science Foundation (grant nr. 20-229845, KS) should read

This work was supported by funds from the Czech Science Foundation (grant nr. 20-22984S, KS)

The original article has been corrected.

